# Expression of the Epidermal Growth Factor Receptor (EGFR) Ligand Heparin-Binding EGF-Like Growth Factor (HB-EGF) in Sinonasal Inverted Papilloma

**DOI:** 10.7759/cureus.96025

**Published:** 2025-11-03

**Authors:** Ryotaro Nakazawa, Takayoshi Ueno, Kazuhira Endo, Satoru Kondo, Hisashi Sugimoto, Tomokazu Yoshizaki

**Affiliations:** 1 Otolaryngology - Head and Neck Surgery, Kanazawa University, Kanazawa, JPN

**Keywords:** adam12, epidermal growth factor receptor (egfr), hb-egf, hif-1α, sinonasal inverted papilloma

## Abstract

Objectives

Nasal sinonasal inverted papillomas (SIP) are benign tumors with malignant potential that may progress to squamous cell carcinoma (SCC). Epidermal growth factor receptor (EGFR) expression is central to the development and malignant transformation of SIP, but the expression of its ligand, heparin-binding EGF-like growth factor (HB-EGF), and the regulators of HB-EGF expression have not been fully investigated. This study aimed to examine the expression of HB-EGF and its regulatory factors in SIP.

Methods

The expression of HB-EGF, EGFR, and phosphorylated EGFR (p-EGFR) was immunohistochemically examined in 32 patients with SIP, 3 with SIP-associated sinonasal squamous cell carcinoma (IPSCC), and 27 with de novo SCC arising in the sinonasal cavity (dnSCC). We also evaluated the expression of HIF-1α, an activator of ADAM12, which promotes the shedding of cytoplasmic membrane-bound HB-EGF from the cell surface.

Results

Among the 32 patients with SIP, most corresponded to Krouse stages T2-T3 and originated mainly in the maxillary or ethmoid sinus. All three cases of IPSCC were of the keratinizing type and classified as T4a. The 27 dnSCC cases were predominantly keratinizing type, arising chiefly in the maxillary sinus, and staged as T3-T4. HB-EGF and ADAM12 were expressed in all SIP and IPSCC cases, whereas the HB-EGF positivity rate was significantly lower in dnSCC (p < 0.05). EGFR and ADAM12 were broadly expressed across groups without significant differences, while p-EGFR expression was significantly higher in SIP and IPSCC compared with dnSCC (p < 0.05). Correlation analysis demonstrated a strong association between ADAM12 and HIF-1α, with weaker positive correlations observed between ADAM12 and HB-EGF, and between HB-EGF and p-EGFR.

Conclusion

HB-EGF expression is a characteristic feature of SIP. ADAM12 and HIF-1α may contribute to the distinctive upregulation of HB-EGF observed in SIP.

## Introduction

Sinonasal inverted papillomas (SIP) are benign epithelial tumors that account for more than 4% of all sinonasal tumors [1]. Treatment of SIP involves surgical resection; however, the recurrence rate is high, ranging from 15% to 23% [2-4]. Additionally, 6.5%-10% of patients may have concurrent squamous cell carcinoma (SCC) arising from SIP (IPSCC) [2,4,5], which distinguishes SIP from typical benign tumors..

The proposed mechanisms underlying the development and malignant transformation of SIP include p53 overexpression and Epstein-Barr virus infection [6]. In 1983, Syriänen HJ et al. reported the presence of human papillomavirus (HPV) in SIP tissues [7]. Since then, various studies have investigated the relationship between SIP and HPV. However, HPV detection rates vary widely, and its association with SIP remains controversial [8,9].

Recently, the epidermal growth factor receptor (EGFR) has attracted attention for its potential involvement in the development and malignant transformation of SIP [6]. Cabal VN et al. reported that EGFR and phosphorylated EGFR (p-EGFR) are expressed in 92% and 54% of SIP, respectively, and that EGFR exon 20 mutations are present in 38% of SIP and 50% of de novo SCC arising in the sinonasal cavity (dnSCC), suggesting that EGFR mutations and phosphorylation-induced activation are closely associated with SIP and IPSCC [10]. Thus, EGFR expression is a common feature of SIP and plays an important role in its malignant transformation.

Heparin-binding EGF-like growth factor (HB-EGF) is a member of the EGF family and is synthesized as a transmembrane precursor molecule called the HB-EGF precursor (pro-HB-EGF). During epithelial repair, HB-EGF is cleaved by specific metalloproteinases into a mature, secreted growth factor called soluble HB-EGF (sHB-EGF), which is subsequently released extracellularly. The soluble form binds to and activates EGFR through paracrine signaling, thereby promoting cell proliferation [11-13].

ADAM12, a member of the a disintegrin and metalloprotease (ADAM) family, shows upregulated expression in breast and lung cancers [14-16]. ADAM12 is capable of shedding pro-HB-EGF. In pituitary adenomas, ADAM12 cleaves ectodomains such as HB-EGF and releases the EGFR ligand, thereby inducing epithelial-mesenchymal transition and cell invasion [17].

Hypoxia-inducible factor (HIF-1α) regulates the cellular response to hypoxia [18]. Wang R et al. reported that hypoxia induces HIF-dependent expression of ADAM12 in breast cancer cells, resulting in the cleavage of the HB-EGF ectodomain [19]. ADAM12, HB-EGF, and HIF-1α are also reportedly involved in the invasion mechanisms of adenoid cystic carcinoma [20].

EGFR and HB-EGF mRNA are highly expressed in SIP compared with normal nasal mucosa [21]. However, to the best of our knowledge, no reports have examined in detail the expression of HB-EGF or its role in SIP. Considering that smoking and chronic inflammation may cause mucosal hypoxia and upregulate HIF-1α expression [22], which could activate the ADAM12-HB-EGF-EGFR signaling pathway, investigating this mechanism may help clarify one reason why smoking is a risk factor for SIP.

Therefore, this study was conducted to examine the expression of HB-EGF and the regulatory factors of HB-EGF expression in SIP and IPSCC, in comparison with dnSCC, and to explore whether these findings may provide insights for future targeted therapeutic strategies.

## Materials and methods

Target patients

Between 1992 and 2022, tissue specimens were obtained from 32 patients with SIP, 3 patients with IPSCC, and 27 patients with dnSCC without a history of SIP. The clinical data for all cases are summarized in Table 1.

**Table 1:** Clinical data. The combined SIP and IPSCC groups were compared with the dnSCC group. Gender and smoking status were analyzed using the chi-square test, while age was analyzed using the t-test; no statistically significant differences were observed. Differences in primary tumor location between groups were also analyzed using the chi-square test, which revealed a statistically significant difference. The Krouse T stage (used for SIP) and the conventional T stage (used for malignant tumors) are based on different staging systems and are therefore not directly comparable; hence, no statistical analysis was performed for these variables. The primary tumor location was determined based on radiological findings. SIP: Sinonasal inverted papilloma; IPSCC: Inverted papilloma-associated squamous cell carcinoma; dnSCC (SNSCC): De novo sinonasal squamous cell carcinoma; χ²: Chi-square; t: Student’s t-test; p: Probability value; NA: Not applicable.

Variable	SIP (n = 32)	SIP + SCC (n = 3)	SNSCC (n = 27)	Test statistic	p-value
Sex, n (%)				χ² = 0.879	0.349
Male	25 (78%)	2 (67%)	17 (63%)		
Female	7 (22%)	1 (33%)	10 (37%)		
Age, years				t = 1.78	0.287
Mean (range)	59 (34-81)	63 (58-68)	65 (42-80)		
Smoking (Brinkman Index)				χ² = 0.147	0.702
≥ 400	12	2 (67%)	13 (48%)		
< 400	20	1 (33%)	14 (52%)		
Primary location, n (%)				χ² = 11.20	< 0.05
Maxillary sinus	14 (44%)	1 (33%)	23 (85%)		
Ethmoid sinus	11 (34%)	1 (33%)	2 (7%)		
Frontal sinus	1 (3%)	0 (0%)	0 (0%)		
Sphenoid sinus	1 (3%)	0 (0%)	0 (0%)		
Nasal cavity	5 (16%)	1 (33%)	2 (7%)		
Krouse T stage, n (%)					
T1	3 (9%)	NA	NA		
T2	9 (28%)	NA	NA		
T3	19 (59%)	NA	NA		
T4	1 (3%)	NA	NA		
T stage, n (%)					
T1	NA	0 (0%)	1 (4%)		
T2	NA	0 (0%)	0 (0%)		
T3	NA	0 (0%)	11 (41%)		
T4a	NA	3 (100%)	12 (44%)		
T4b	NA	0 (0%)	3 (11%)		

Immunohistochemistry (IHC) 

Protein expression was analyzed by IHC on 4-µm sections of individual tissue blocks from SIP and dnSCC cases. The antibodies used for IHC included anti-HB-EGF monoclonal antibody (mAb) (Medical & Biological Laboratories (MBL) Co., Ltd., Nagoya, Japan), anti-ADAM12 (Proteintech, Manchester, UK), anti-EGFR (Abcam, Shanghai, China), anti-p-EGFR (Cell Signaling Technology (CST), Massachusetts, USA), and anti-HIF-1α (Santa Cruz Biotechnology (SCBT), Texas, USA).

Paraffin-embedded sections were deparaffinized and treated with 10 mM citrate buffer (pH 6.0) at 98 °C for 30 min using a hot water dispenser. Endogenous peroxidase activity was blocked by incubation with 3% H₂O₂ in phosphate-buffered saline (PBS) at room temperature for 10 min. Sections were then incubated with a protein block (Dako) at room temperature for 30 min, followed by incubation with each primary antibody in PBS overnight at 4 °C, and with secondary antibodies (Dako) for 1 h at room temperature. Staining was visualized using a diaminobenzidine (DAB) substrate, and sections were lightly counterstained with Mayer’s hematoxylin.

Immunostained tumor sections were evaluated by investigators blinded to the clinical data. Tumor tissues were randomly observed at 400× magnification, and the percentage of positive cells was calculated.

IHC for EGFR and p-EGFR was considered positive when moderate to strong membranous staining was observed in more than 10% of tumor cells; cases that did not meet this criterion were regarded as negative, based on Menendez M et al. [23].

Similarly, IHC for HB-EGF, ADAM12, and HIF-1α was considered positive when more than 10% of tumor cells showed nuclear or cytoplasmic staining.

Statistical analysis

R version 4.2.2 was used to analyze associations between clinical and immunohistochemical factors using the chi-square test. Differences in primary tumor location among groups were also evaluated using the chi-square test. Age comparisons between two groups were performed using the t-test. Correlations were assessed using Pearson’s correlation analysis in the same software. p-values < 0.05 were considered statistically significant.

## Results

Of the 32 patients with SIP, 7 were women (22%) and 25 were men (78%). The mean age was 59 years (range, 34-81). According to the Krouse classification, there were 3 T1 (9%), 9 T2 (28%), 19 T3 (59%), and 1 T4 (3%) tumors. Overall, 14 tumors originated in the maxillary sinus (44%), 11 in the ethmoid sinus (34%), 1 in the frontal sinus (3%), 1 in the sphenoid sinus (3%), and 5 in the nasal cavity (16%). All IPSCC cases (n = 3; 1 woman and 2 men; mean age, 63 years (range, 58-63); all T4a) were of the keratinizing type. Of the three cases, one tumor arose in the maxillary sinus, one in the ethmoid sinus, and one in the nasal cavity.

All 27 cases of dnSCC (10 women (37%) and 17 men (63%); mean age, 65 years (range, 42-80)) were of the keratinizing type. Among these, 23 tumors (85%) originated in the maxillary sinus, 2 (7%) in the ethmoid sinus, and 2 (7%) in the nasal cavity. One case was stage T1 (4%), none were T2, 11 were T3 (41%), 12 were T4a (44%), and 3 were T4b (11%). Detailed clinical data are provided in Table 1.

IHC analysis

HB-EGF showed positive staining in all SIP cases, with nuclear and cytoplasmic localization. All three IPSCC cases were also positive. In contrast, only 14 (52%) dnSCC cases were positive. The positivity rate for dnSCC was significantly lower than those for SIP and IPSCC (p < 0.05).

Similar to HB-EGF, ADAM12 was positive in all SIP cases, with nuclear and cytoplasmic staining. All three IPSCC cases were also positive, as were 26 of 27 (96%) dnSCC cases; there was no significant difference between the groups (p = 0.896).

EGFR showed positive plasma membrane staining in 31 of 32 SIP cases (97%). EGFR was positive in all IPSCC cases and in 22 of 27 (81%) dnSCC cases; the difference between the groups was not statistically significant (p = 0.102).

For p-EGFR, 22 of 32 SIP cases (69%) showed positive plasma membrane staining. All IPSCC cases also showed positive staining, with greater intensity than that observed in SIP. Among dnSCC cases, 10 of 27 (37%) were positive; this rate was significantly lower than that for SIP and IPSCC combined (p < 0.05).

Overall, 29 of 33 SIP cases (88%), all IPSCC cases, and 22 of 27 dnSCC cases (81%) were positive for HIF-1α. The difference between SIP and dnSCC cases was not statistically significant (p = 0.24).

**Figure 1:**
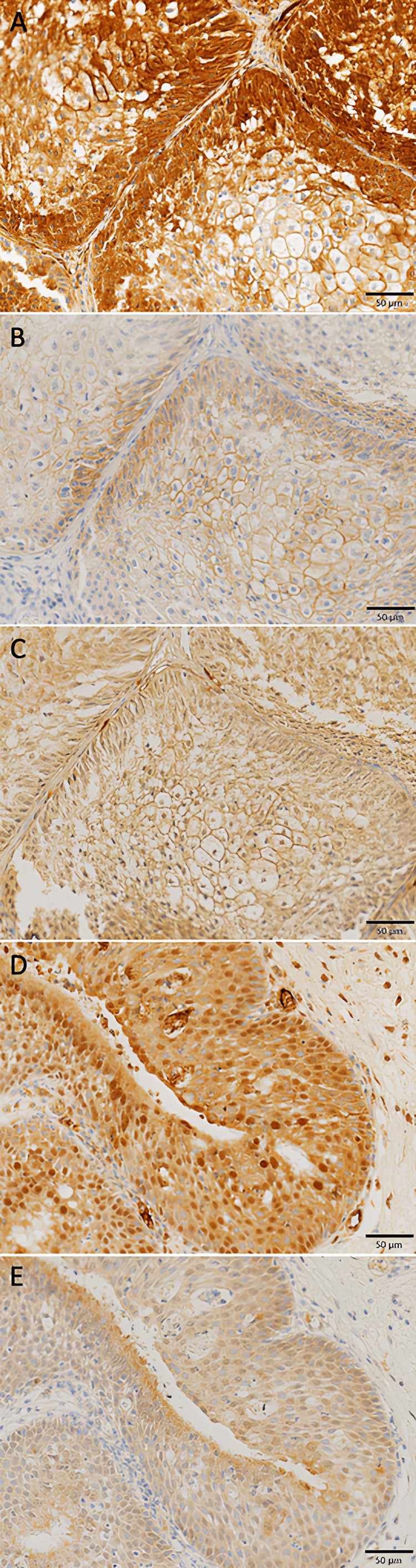
Photomicrographs of immunohistochemical expression of each antibody in SIP (20×). (A) EGFR: Staining of the cell membrane is observed. (B) p-EGFR: As with EGFR, staining of the cell membrane is observed. (C) HB-EGF: Staining of the cell membrane and nuclei is observed. (D) ADAM12: Staining of the plasma membrane, cytoplasm, and nuclei is observed. (E) HIF-1α: Staining of the cytoplasm and nuclei is observed. SIP: Sinonasal inverted papilloma; EGFR: Epidermal growth factor receptor; p-EGFR: Phosphorylated epidermal growth factor receptor; HB-EGF: Heparin-binding EGF-like growth factor; ADAM12: a disintegrin and metalloprotease 12; HIF-1α: Hypoxia-inducible factor 1-alpha.

The results of all IHC evaluations are summarized in Table 2. Representative images of SIP immunostaining are presented in Figure 1.

**Table 2:** Immunohistochemical staining results of HB-EGF, ADAM12, EGFR, p-EGFR, and HIF-1α. The combined SIP and IPSCC groups were compared with the dnSCC group. HB-EGF and p-EGFR were significantly more highly expressed in SIP compared with dnSCC. ADAM12 and EGFR were both expressed at high rates, with no significant difference between the two groups. HIF-1α expression was also not significantly different between the groups, although it tended to be higher in SIP than in dnSCC. Differences in expression between groups were evaluated using the chi-square test. SIP: Sinonasal inverted papilloma; IPSCC: Inverted papilloma-associated squamous cell carcinoma; dnSCC: De novo sinonasal squamous cell carcinoma; HB-EGF: Heparin-binding EGF-like growth factor; ADAM12: a disintegrin and metalloprotease 12; EGFR: Epidermal growth factor receptor; p-EGFR: Phosphorylated epidermal growth factor receptor; HIF-1α: Hypoxia-inducible factor 1-alpha.

Marker	SIP (n = 32)	SIP + SCC (n = 3)	SNSCC (n = 27)	Test statistic	p-value
HB-EGF	32 (100%)	3 (100%)	14 (52%)	χ² = 20.57	< 0.05
ADAM12	32 (100%)	3 (100%)	25 (93%)	χ² = 0.832	0.362
EGFR	31 (97%)	3 (100%)	23 (85%)	χ² = 1.548	0.213
p-EGFR	24 (75%)	3 (100%)	13 (48%)	χ² = 4.402	< 0.05
HIF-1α	30 (94%)	3 (100%)	22 (81%)	χ² = 1.380	0.24

**Figure 2:**
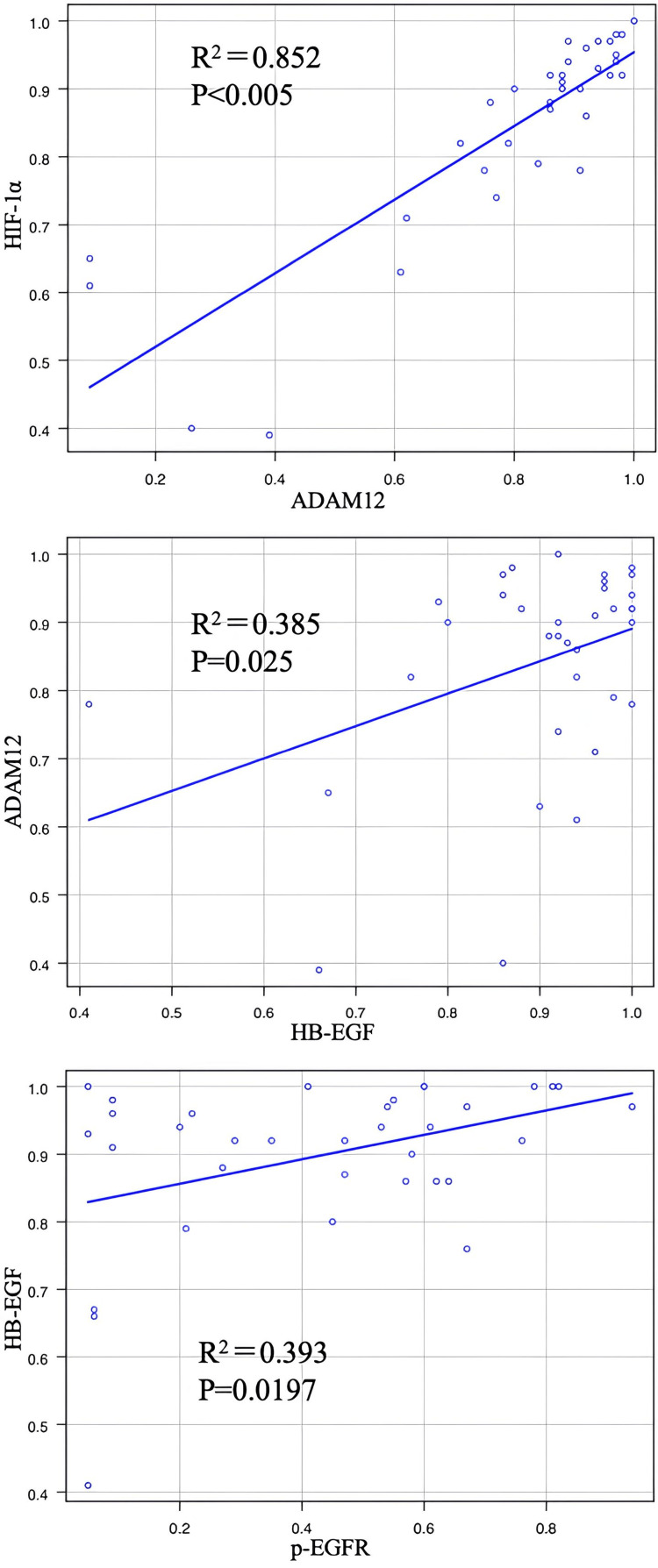
Correlation between HIF-1α, ADAM12, HB-EGF, and p-EGFR expression. A strong positive correlation is observed between HIF-1α and ADAM12. Weak positive correlations are observed between ADAM12 and HB-EGF, and between HB-EGF and p-EGFR. HIF-1α: Hypoxia-inducible factor 1-alpha; ADAM12: a disintegrin and metalloprotease 12; HB-EGF: Heparin-binding EGF-like growth factor; p-EGFR: Phosphorylated epidermal growth factor receptor.

Next, the relationships between immunohistochemical markers were analyzed. A strong positive correlation was observed between ADAM12 and HIF-1α (γ = 0.852, p = 8.46 × 10⁻¹¹). ADAM12 and HB-EGF showed a weak positive correlation (γ = 0.385, p = 0.025), while HB-EGF and p-EGFR also showed a weak positive correlation (γ = 0.393, p = 0.0197) (Figure 2).

## Discussion

Activation of EGFR by phosphorylation is associated with tumor cell proliferation and invasion [24]. In this study, the IHC results revealed strong expression of HB-EGF and ADAM12 in all SIP tissues. Additionally, EGFR, to which the ectodomain of HB-EGF binds, was highly expressed in almost all SIP cases, consistent with previous reports. The positivity rate of p-EGFR was approximately 70%, also in line with previous studies [10,21,23].

These findings suggest that ADAM12 mediates the shedding of the ectodomain of pro-HB-EGF in SIP, which then binds to EGFR and activates it, thereby establishing a signaling pathway that contributes to SIP tumor growth. Our results also suggest that HB-EGF and ADAM12 expression are closely associated in SIP.

This pathway of EGFR activation has already been demonstrated in breast cancer, and HIF-1α reportedly induces HB-EGF shedding by ADAM12 [19]. In our study, we confirmed the expression of HIF-1α, which is responsible for inducing ADAM12 expression, using the same tissue samples. Overall, 88% of SIP specimens showed HIF-1α positivity, and all IPSCC specimens strongly expressed HIF-1α. Another study examining HIF-1α expression in SIP also suggested HIF-1α upregulation in SIP [25].

**Figure 3:**
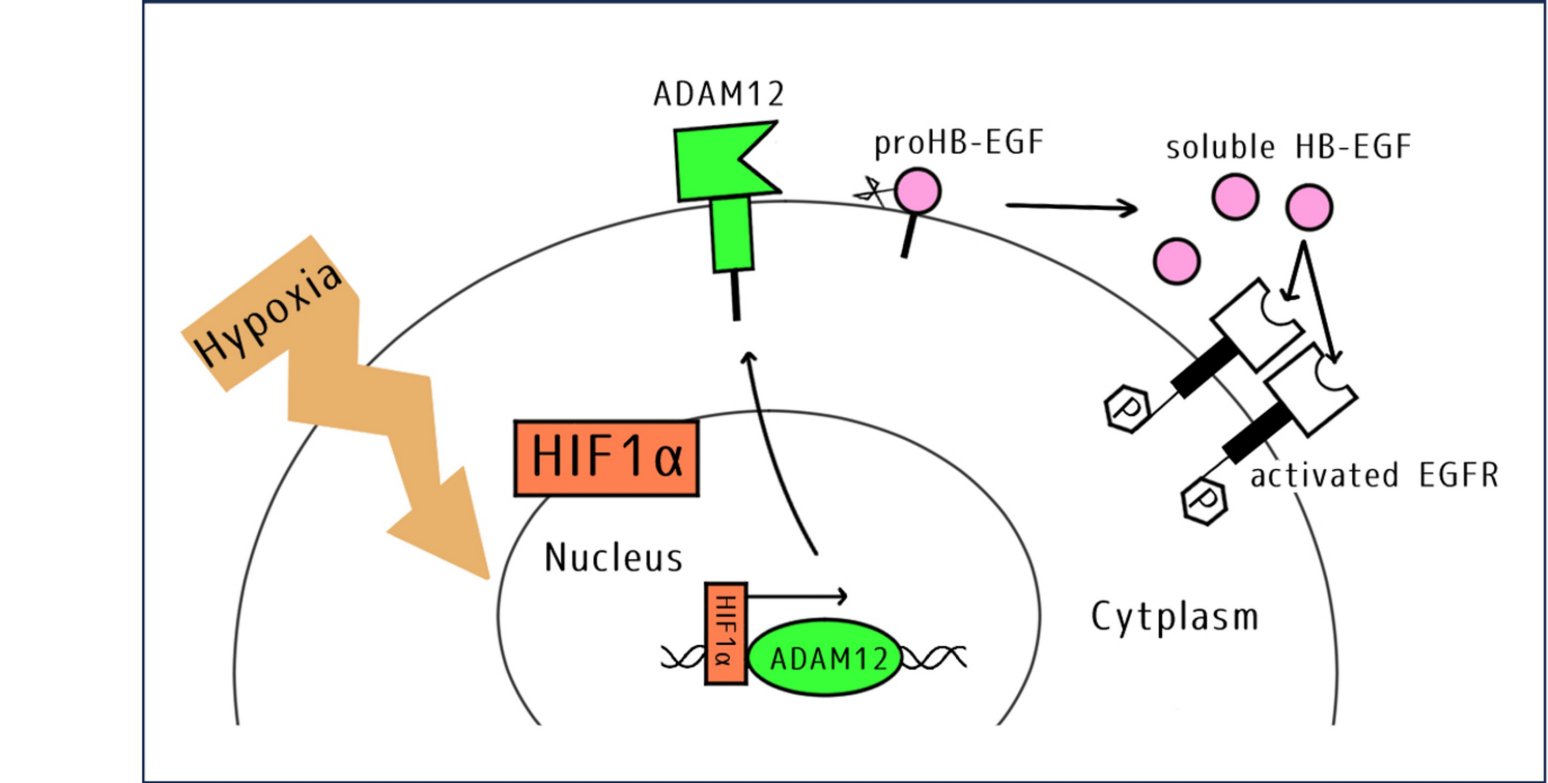
Proposed model for tumor cell proliferation through shedding of HB-EGF by ADAM12 under hypoxic conditions. Image credits: Ryotaro Nakazawa. In cells located within hypoxic regions, levels of HIF-1α are stabilized, and increased HIF-1α expression induces ADAM12, whose activity promotes ectodomain shedding of pro-HB-EGF. The released HB-EGF binds to EGFR) on the same, adjacent, or distant cells, thereby promoting tumor cell proliferation. HIF-1α: Hypoxia-inducible factor 1-alpha; ADAM12: a disintegrin and metalloprotease 12; HB-EGF: Heparin-binding EGF-like growth factor; pro-HB-EGF: Precursor form of heparin-binding EGF-like growth factor; EGFR: Epidermal growth factor receptor.

Analysis of the IHC results revealed a strong positive correlation between ADAM12 and HIF-1α, suggesting that ADAM12 may be induced by HIF-1α in SIP. The same correlation was also examined in dnSCC; however, no positive correlation was observed (correlation coefficient = 0.33), and no clear association between ADAM12 and HIF-1α was identified in dnSCC tissues. Thus, it is possible that ADAM12 is induced by HIF-1α in SIP, as in breast cancer, and that this induces the EGFR activation pathway (Figure 3).

HIF-1α, which is the initiating factor of this cell proliferation pathway, is a regulator of the cellular response to hypoxia. Previous reports [22] have linked smoking to sinusitis, suggesting that smoking induces an inflammatory response in the nasal mucosa, resulting in tissue hypoxia that may promote HIF-1α expression. Traditionally, smoking has been suspected as one of the etiological factors of SIP [26], and Hong SL et al. [27] reported that smoking is associated with the recurrence and malignant transformation of SIP. In addition to smoking, chronic inflammation [28,29], which is often associated with cancer development in general [30], plays a role in the development and progression of SIP. Considering these findings, it is possible that the cell proliferation pathway initiated by HIF-1α contributes to the development, recurrence, and malignant transformation of SIP.

In this study, we have shown that one of the cell growth pathways in SIP may be driven by HB-EGF-mediated EGFR activation. Additionally, a comparison with dnSCC showed that SIP exhibits higher expression of HB-EGF, and this proliferative pathway appears to be a distinctive feature of SIP. Indeed, Udager AM et al. reported that treatment of IPSCC cells with irreversible EGFR inhibitors inactivates EGFR signaling and inhibits cell growth [31]. Considering the results of this study, it is likely that EGFR activation also contributes to SIP growth, and that EGFR inhibitors or molecularly targeted drugs against HB-EGF may suppress tumor proliferation. If such inhibitory effects are confirmed, these therapies could be valuable for treating SIP cases in which surgical intervention is not feasible.

A SIP cell line has not yet been established; therefore, we could not perform experiments involving drug administration in this study. If a SIP cell line is developed in the future, it will be necessary to validate our findings by, for example, inhibiting HB-EGF function. Administration of an antibody targeting HB-EGF in a SIP cell line is expected to exert an inhibitory effect on cell proliferation.

With respect to SIP, EGFR mutations and p-EGFR expression are mutually exclusive [10]. There are also reports suggesting that SIP harboring EGFR mutations have a lower risk of progression to SCC [32]. Hence, it is important to examine the activation of downstream signaling pathways in both EGFR-mutant and p-EGFR-positive SIP. If these mechanisms activate a common pathway, the downstream molecules may represent potential therapeutic targets for SIP. We intend to confirm EGFR mutations in the specimens used in this study and compare them with p-EGFR expression results in a larger number of IPSCC cases to validate previous reports and clarify the risk of progression to SCC with and without p-EGFR expression.

This study has several limitations. First, it was a retrospective analysis with a relatively small number of cases, which may limit the generalizability of the results. Compared with a prospective design, the amount and consistency of available clinical data were limited, and detailed information regarding comorbidities or coexisting tumors that might have contributed to tissue hypoxia was not available for all patients. Second, we were unable to perform functional assays because a SIP cell line has not yet been established. Third, although we identified correlations among HB-EGF, ADAM12, and HIF-1α expression, the causal relationships among these molecules were not directly demonstrated. Future studies using cell lines or animal models will be necessary to confirm the functional roles of these factors in SIP tumorigenesis.

## Conclusions

Our study demonstrates that HB-EGF is highly expressed in SIP and, through its role as a ligand for EGFR, may drive a distinct proliferative pathway contributing to tumor growth. We also found that ADAM12 and HIF-1α are closely associated with this mechanism, suggesting that hypoxia- and inflammation-related factors may regulate HB-EGF-mediated EGFR activation. These findings highlight the biological uniqueness of SIP compared with dnSCC and provide a rationale for exploring EGFR- or HB-EGF-targeted therapeutic strategies, particularly in cases where surgical intervention is not feasible or the risk of recurrence is high.
